# T1期非小细胞肺癌纵隔淋巴结转移规律及清扫方式研究进展

**DOI:** 10.3779/j.issn.1009-3419.2015.01.06

**Published:** 2015-01-20

**Affiliations:** 100021 北京，北京协和医学院中国医学科学院肿瘤医院胸外科 Department of Toracic Surgery, Cancer Hospital, Peking Union Medical College and Chinese Academy of Medical Sciences, Beijing 100021, China

**Keywords:** 肺肿瘤, 淋巴结转移, 纵隔淋巴结清扫, 纵隔淋巴结取样, Lung neoplasms, Lymph node metastasis, Mediastinal lymph node dissection, Mediastinal lymph node sampling

## Abstract

肺癌是我国发病率和死亡率最高的恶性肿瘤。非小细胞肺癌（non-small cell lung cancer, NSCLC）约占肺癌80%。临床上，早期NSCLC以手术治疗为主要治疗方式，淋巴结分期及手术中清扫程度直接影响着患者的预后。不同肺叶原发NSCLC的淋巴结转移区域存在一定规律。解剖性肺叶切除加系统性淋巴结清扫一直以来被认为是NSCLC的标准手术方式，但近年来T1期NSCLC手术中纵隔淋巴结清扫的程度存在较大争议，选择性淋巴结清扫已逐渐被大多数学者所重视。

## Abstract (en)

Lung cancer ranks the frst position in morbidity and mortality among all malignances in China. Nonsmall cell lung cancer (NSCLC) accounts for nearly 80% of all lung malignancies. Surgical resection is still the current major treatment method for early stage NSCLC. Lymph node stages together with the extent of lymph node dissection directly a?ect the prognosis. Te site of primary lung carcinoma may a?ect the route and status of lymph node metastasis. Up to now, anatomical lobectomy and mediastinal lymph node dissection is deemed to be the standard surgical treatment for NSCLC. However, it is still controversial in the extent of lymph node dissection for T1 NSCLC. More and more surgeons intend to perform selective mediastinal lymph node dissection instead of complete mediastinal lymph node dissection for T1 NSCLC in recent years.

目前，肺癌已成为我国发病率和死亡率最高的恶性肿瘤，其中非小细胞肺癌（non-small cell lung cancer, NSCLC）约占肺癌80%^[1]^。NSCLC已成为全球范围内癌症死亡的主要原因^[2]^。在中国，无论是男性还是女性，城市或乡村，肺癌死亡率位居癌症死亡的首位^[3]^。肺癌早期诊断、准确分期及适当的治疗方式对于提高肺癌患者生存率至关重要。随着影像学技术的飞速发展及人们健康意识的不断提高，早期肺癌的发现比率正逐年增加。

T1期肺癌是指肿瘤最大直径≤3 cm，周围由肺或脏层胸膜包绕，支气管镜下肿瘤未超出叶支气管（即未侵犯左右主支气管），最新的肿瘤-淋巴结-转移（tumor-node-metastasis, TNM）分期又将T1期肺癌分为T1a（肿瘤最大径≤2 cm）及T1b（2 cm < 肿瘤最大径≤3 cm）^[4]^。T1期病变纵隔淋巴结转移是影响NSCLC患者预后的一个重要因素，正确评估早期NSCLC患者淋巴结转移情况对于选择适当的治疗方案起着关键作用，因为早期肺癌行手术治疗时清扫转移淋巴结对提高患者总生存率及术后无病生存率起着关键作用^[5]^。然而目前，术前很难明确判断NSCLC患者纵隔淋巴结受累情况，因此，临床上NSCLC手术过程中纵隔淋巴结清扫方式一直以来存在很大争议。现总结国内外近几年来相关研究，并就T1期NSCLC淋巴结病变的诊断、不同部位原发病灶纵隔淋巴结转移规律及手术中淋巴结清扫方式进行综述。

## 1 术前淋巴结病变的评估方法及效果

### 1.1 计算机断层扫描（computed tomography, CT）

胸部增强CT对于早期肺癌的诊断发挥着重要的作用，同时CT也是目前临床上评估NSCLC淋巴结转移情况并进行临床分期的主要依据，国内外学者大都把淋巴结短径≥1 cm作为淋巴结转移的标准^[6-8]^。但以该诊断标准判断纵隔淋巴结病变的敏感性及特异性均较低，德国学者研究报道^[7]^病理确诊无淋巴结转移NSCLC患者中，77%患者至少有一个淋巴结直径 > 1 cm，而确定为淋巴结转移的患者中，12%未发现直径超过1 cm的淋巴结，因此认为CT通过淋巴结的直径大小来判断是否为转移性淋巴结并非可靠标准。Silvestri等^[6]^对43项相关研究进行*meta*分析发现其敏感性和特异性分别为51%和86%。

### 1.2 正电子发射计算机断层显像（positron emission tomography CT, PET-CT）

PET是一种功能性的显像模式，可通过判断肿瘤组织代谢活性来发现早期转移病变。临床上常以标准摄取值（standardized uptake value, SUV） > 2.5来区别肿瘤组织与正常组织，并通过SUV值预测肿瘤的生存率及治疗效果^[9]^。Darling等^[1]^研究发现PET-CT在诊断纵隔淋巴结转移时敏感性约为70%，特异性则高达94%。然而通过SUV值判断纵隔淋巴结转移存在较高假阳性率（肺部肉芽肿性病变及炎性病变均可引起SUV值升高），未进行病理确诊时，临床上可能做出错误的判断，使一部分患者失去手术机会。近年来，Cerfolio等^[2]^对153例经PET-CT检查诊断为纵隔淋巴结转移阴性的的患者进行前瞻性研究发现PET-CT诊断为N1的患者中17.6%行纵隔镜检查明确为N2淋巴结阳性，23.5%的N1期病变行内镜超声引导细针穿刺检查明确为N2淋巴结阳性。

### 1.3 纵隔镜活检

经纵隔镜行纵隔淋巴结活检是目前对NSCLC患者进行纵隔淋巴结分期的金标准^[10, 11]^。纵隔镜可完成2区、4区、3区及7区前半部分等前纵隔区域的淋巴结活检，纵隔镜行淋巴结活检在诊断纵隔淋巴结病变时敏感性及特异性分别高达81%及100%^[12]^。纵隔镜淋巴结活检亦存在其局限性，即不能进行5区、6区、8区、9区及7区后半部分的淋巴结取样活检术。而且，经纵隔镜检查行淋巴结活检作为一项侵入性检查具有一定的风险，其并发症发生率、死亡率分别为2%和0.08%^[13]^。由于经支气管镜超声引导纵隔淋巴结针吸活检术创伤更少，已经显现逐渐替代纵隔镜的趋势。

### 1.4 超声支气管镜穿刺和内镜超声引导细针穿刺活检（endobronchial ultrasound-transbronchial needle aspiration, EBUS-TBNA）

EBUS-TBNA是指在支气管镜前端微型超声探头的引导下行实时经支气管壁对壁外组织进行穿刺活检。Yasufuku等^[14]^对EBUS-TBNA与纵隔镜用于肺癌纵隔淋巴结分期进行前瞻性对照研究发现，EBUS-TBNA的敏感性、阴性预测价值和准确性分别为81%、91%和93%，纵隔镜检查的敏感性、阴性预测价值及准确率各为79%、90%和93%，而两种检查方式的特异性及阳性预测价值均为100%，两种方式评价患者淋巴结分期无统计学差异（*P*=0.78）。近年来其他学者也报道EBUS-TBNA与纵隔镜在肺癌外科分期方面效果相近^[15]^，但与纵隔镜相比，EBUS-TBNA可在局麻下进行，并发症更少，也更加安全、方便、经济，已逐渐取代纵隔镜淋巴结活检^[14, 16]^。

内镜超声引导细针穿刺活检（endoscopic ultrasound-guided fine-needle aspiration, EUS-FNA）是经食道超声引导，直视行纵隔淋巴结穿刺活检，能够进行5区、7区、8区、9区等纵隔镜不易到达区域的淋巴结活检，其在临床上应用已超过20余年^[17, 18]^。EUS-FNA评价NSCLC纵隔淋巴结病变安全且有效，*meta*分析^[19]^显示诊断N2病变的敏感性及特异性分别为83%，97%，假阴性率为22%。

### 1.5 电磁导航支气管镜（electromagnetic navigation bronchoscopy, ENB）

ENB是一项类似于全球定位导航系统（global positioning system, GPS）的新型技术，能够指导支气管镜检查仪器到达肺内病变靶区域及纵隔内病变淋巴结进行活检、刷片及经支气管细针穿刺等操作，对于CT检查无法明确病变性质而常规支气管镜检查无法到达的外周肺部病变，ENB能够明显提高病变诊断率并降低穿刺引起的气胸、低氧血症、出血等并发症^[20-22]^。ENB系统主要包括虚拟支气管镜计划软件、能释放低频电磁波的定位板、8个方向可操纵导管以及带有可精确跟踪电磁场位置和方向传感器的可定位探针组成；通过获取高质量CT扫面图像，预先设定厚层函数并载入计划软件后重建图像，插入定位导管及导丝并对与CT显示相匹配的重要解剖位置进行标记，通过屏幕显示图像引导操作者沿设定路线直达靶目标并显示探头与目标的距离，锁定定位导管插入活检工具进行活检等步骤完成病变靶区的活检^[20, 22]^。Gildea等^[20]^通过前瞻性研究发现ENB技术对周围型肺癌及纵隔淋巴结的确诊率分别高达71%和100%，并发现ENB技术的高确诊率与肺及淋巴结病变的大小及位置无明显相关性（*P* > 0.05）。

## 2 T1期NSCLC的淋巴结转移规律研究进展

### 2.1 生理性肺淋巴液引流规律的研究

研究者们^[23, 24]^曾通过灌注技术从解剖学上研究肺的淋巴引流方式，然而这仅能够发现浅表的胸膜下淋巴结引流途径，却难以发现肺内淋巴结引流，同时注射速率也能影响其研究结果。组织胞浆菌病^[25]^是一种原发性真菌病，常侵犯到肺引起原发部位实变，并累及肺门及纵隔淋巴结引起相应淋巴结钙化，Takahashi等^[26]^通过分析该病CT表现的分析发现肺部病变的淋巴液经肺门到纵隔淋巴结引流遵循一定规律，并设想该规律为肺部淋巴液在生理情况下的引流方式：右肺上叶淋巴液易引流至4R区、肺门、肺内淋巴结，右中叶淋巴液易引流至7区、4R区、肺门及肺内淋巴结，右下叶淋巴液易引流至7、9、8区、肺门及肺内淋巴结，左上叶肺淋巴液易引流至5、7、4L区、肺门及肺内淋巴结，左下肺淋巴液易引流至7、8、9区、肺门及肺内淋巴结；同时该研究还发现，肺部病变出现纵隔淋巴结跳跃式转移（是指无肺内及肺门淋巴结转移，但出现纵隔及远处淋巴结病变）表现如下规律：右上叶肺病变易出现4R区跳跃式淋巴结病变，右下叶肺病变易发生在8区跳跃式淋巴结病变，左上叶肺病变易出现5区跳跃式淋巴结病变，左下叶肺易出现9区跳跃式淋巴结病变；因此作者认为其研究结果对NSCLC淋巴结引流方式具有预测意义。但是，一些研究^[27]^证明肺的淋巴引流系统在解剖结构上存在很大的变异性，认为肺癌的淋巴结转移方式可能无法预测。

### 2.2 肺癌原发部位与淋巴结引流

NSCLC纵隔淋巴结转移对于患者准确的临床分期及制定相应的治疗方案至关重要，对手术清扫方式和确定放射治疗靶区也都有非常重要的意义，尤其对于T1期NSCLC更为明显。近年来，国内外学者进行了大量的研究寻求不同肺叶NSCLC的纵隔淋巴结转移规律。

希腊学者^[28]^回顾性研究557例术前诊断为淋巴结病变阴性的NSCLC且均行手术治疗加系统性纵隔淋巴结清扫的患者，其研究显示不同肺叶原发NSCLC纵隔淋巴结转移表现出一定规律：右上叶肺癌主要转移至4R区、肺叶间和肺门淋巴结，右肺中叶癌主要转移至7、4R区、肺门和肺叶间淋巴结，右肺下叶癌主要转移至7区、肺门和肺叶间淋巴结，左肺上叶癌主要转移至5区、肺门和肺叶间淋巴结，左肺下叶癌主要转移至7、8、9区、肺门和肺叶间淋巴结。而且相关研究^[28-30]^关于NSCLC原发部位与淋巴结转移的关系曾有类似的报道。Cerfolio等^[31]^及其他学者^[32, 33]^对不同肺叶原发NSCLC发生淋巴结转移的规律的研究结果与以上类似，同时还发现与原发于左侧的肺癌相比，原发于右侧肺叶的肺癌更容易发生纵隔淋巴结转移（*P*=0.02）。

国内学者^[34]^研究发现对于直径≤3 cm的周围型NSCLC，肿瘤直径越大，其纵隔淋巴结转移率越高，上肺叶肺癌主要转移至上纵隔淋巴结，下肺叶肺癌则隆突下及上纵隔均可转移，转移的纵隔淋巴结左肺主要分布在第5、6、7组、肺门及肺内淋巴结，右肺主要分布在3、4、7组、肺门及肺内淋巴结，并指出术中应该重点清扫以上区域淋巴结。

### 2.3 肺癌病理类型与淋巴结引流

Watanabe等^[35]^研究发现肺腺癌比鳞癌更容易发生纵隔淋巴结转移，即使直径≤2 cm的腺癌也易发生肺门及纵隔淋巴结转移的。Libshitz等^[36]^研究结果同样显示腺癌易引起纵隔淋巴结转移，而且其发生跳跃式淋巴结转移的比率也高于其他类型的肿瘤。早期肺腺癌还与淋巴结微转移的发生密切相关^[37]^。

### 2.4 肺癌影像学特征与淋巴结引流

Matsuguma等^[38]^对383例高分辨率CT上表现实性占位病变直径≤3 cm、磨玻璃样改变成分不同的NSCLC进行回顾性研究，发现在实性占位病变直径相同时，患者CT表现磨玻璃改变成分 > 50%组肿瘤侵袭淋巴结、胸膜及周围血管的概率及手术后肿瘤复发率明显低于磨玻璃改变成分 < 50%组。其他研究者^[39-43]^进行肺腺癌影像学特征与预后的研究也认为CT上磨玻璃改变成分高的患者不易出现淋巴结及周围组织的转移，预后较好。

### 2.5 肺癌病灶大小与淋巴结转移

谢等^[34]^对直径≤3 cm的周围型肺癌淋巴结转移规律研究发现随着肿瘤直径的增加，纵隔淋巴结转移率明显增加，肿瘤直径在0 cm-1 cm、1 cm-2 cm和2 cm-3 cm时，其N2组淋巴结转移率分别为0、10.5%和29.7%，差异有统计学意义（*P*=0.003)。日本学者^[44]^回顾性研究225例直径≤2 cm的周围型肺癌系统性淋巴结清扫后病理分期发现，直径≤1 cm组病例各组织学类型周围型肺癌均未发生纵隔淋巴结转移，而直径1 cm-2 cm组肺癌患者中有22.3%的肺腺癌出现纵隔淋巴结转移，这也证明肺癌纵隔淋巴结转移的概率随肿瘤直径的增大而增加。

### 2.6 淋巴结微转移与孤立肿瘤细胞转移

淋巴结微转移^[45]^主要是指直径≤2 mm的转移性淋巴结病变，可通过病理学HE染色、免疫组化及聚合酶链反应等方式检测；孤立肿瘤细胞转移（isolated tumor cells, ITC）是指通过免疫组化、聚合酶链反应及其他分子生物学方法检测出的直径≤0.2 mm单个肿瘤细胞或细胞团。有研究报道早期NSCLC出现微转移及ITC的概率可高达20%-30%^[46, 47]^，同时大量研究^[3, 48-51]^显示淋巴结微转移及ITC对早期NSCLC有治疗学意义，明显影响患者预后。相反，Rena等^[37]^研究显示对于临床分期为Ⅰ期NSCLC患者，伴有微转移或ITC组对比不伴微转移或ITC组2年生存率分别为79%、81%，5年生存率均为64%，并认为微转移及ITC对于临床Ⅰ期NSCLC预后（复发率、总生存率，无病生存率）无明显影响。其研究还发现淋巴结微转移主要与肿瘤病理类型相关——腺癌最容易引起淋巴结微转移及ITC，而与肿瘤T分期无直接相关性。

## 3 T1期NSCLC淋巴结清扫方式研究进展

### 3.1 系统性淋巴结清除术

系统性淋巴结清除指将相应肺叶间、肺门淋巴结，纵隔范围内所有的淋巴结及周围脂肪组织行一并切除^[52]^。右侧系统性清除应包括上纵隔4R、2R、3a、3p区域及下纵隔7、8、9区的淋巴结及隆突下至横隔区域的脂肪组织，相应肺叶间及肺门淋巴结及脂肪组织；而左侧系统性清除应包括相应肺叶间及肺门淋巴结及脂肪组织，上纵隔4L、5区及6区淋巴结组织及下纵隔7、8、9区的淋巴结及隆突下至横隔区域的脂肪组织。目前主张行系统性纵隔淋巴结清扫术的理由主要包括提供更为准确的病理分期和提高患者生存率。而不主张进行系统性淋巴结清扫的理由主要包括手术时间延长，术中及术后并发症增多，国内学者^[53]^、国外Ishiguro等^[54]^研究均证明进行系统性淋巴结清扫时平均手术时间更长、手术损伤更大、术中出血更多、患者住院时间更长。Okada等^[55]^回顾性研究发现系统性淋巴结清扫与选择性淋巴结清扫相比，术后并发症发生率分别为17.3%和10.1%（*P*=0.005）。

### 3.2 选择性淋巴结清除术

选择性淋巴结清除术，又称肺叶特异性淋巴结清扫，是指依据肺癌原发病变部位不同，系统地对相应肺叶间及肺门淋巴结、相应容易发生转移的纵隔区域内的淋巴结及周围脂肪组织进行清除^[52]^。Naruke等^[32]^和Ichinose等^[56]^就各肺叶淋巴结引流方式研究认为，对于T1期周围型鳞癌根据原发肺叶病变部位行选择性淋巴结清扫适用，其指出对于经术中冰冻证实相应肺叶间淋巴结及肺门淋巴结阴性后，依病变原发肺叶位置至少行三个纵隔区域淋巴结清扫及切除至少6个淋巴结行病理检查，并总结出各肺叶病变应清扫如下区域淋巴结：右上叶及右中叶病变：2R、4R、7区；右下叶病变：4R、7、8、9区；左上叶病变：5、6、7区；左下叶病变：7、8、9区。相关研究证明肺叶特异性淋巴结清扫术后出现纵隔淋巴结病变未被清除的概率非常低（< 5%）^[57, 58]^。

### 3.3 淋巴结取样

淋巴结取样是根据术前影像学分析及术中观察，仅依据视觉及触觉切除可能为转移病变的淋巴结，选择性淋巴结取样是指切除术前和术中表现为可疑恶性的淋巴结，系统性淋巴结取样指外科医师术前根据影像学表现及淋巴结转移规律对特定组别淋巴结进行切除行病理检查^[52]^。Gajra等^[59]^认为为了确保术后病理分期的准确性，系统性淋巴结取样至少应该切取不同淋巴结区域6个以上淋巴结。

### 3.4 扩大淋巴结清扫

扩大淋巴结清扫是指通过胸骨切开及颈部切口清扫双侧纵隔及颈部淋巴结及周围脂肪组织^[52]^，该方式创伤大、手术时间长、临床少用。

### 3.5 淋巴结清扫方式的比较

解剖性肺叶切除加系统性淋巴结清扫一直以来被认为是临床Ⅰ期、Ⅱ期NSCLC的标准手术方式。目前临床上对系统性N2淋巴结清扫的长期预后作用及围手术期并发症的问题争议颇大，越来越多的证据显示了选择性淋巴结清除术具可行性及有效性^[60]^。近年来，国内外临床医生对T1期NSCLC手术过程中纵隔淋巴结的清扫方式进行大量研究，胸外科医生在NSCLC手术中N2淋巴结处理策略上存在截然不同的意见。

国内吴等^[61]^研究对532例临床І期-Шa期NSCLC进行随机对照研究，发现系统性淋巴结清除组平均生存时间明显高于淋巴结取样组（43个月*vs* 32个月），行系统性淋巴结清除术后，临床І期、临床Ⅱ期、临床Ⅲa期各组患者的5年生存率也明显高于淋巴结取样组。国内学者^[53]^研究报道直径在2 cm-3 cm间的Ia期NSCLC经系统性淋巴结清除组5年生存率和无病生存率明显高于系统性淋巴结取样组，并认为早期NSCLC手术中行系统性淋巴结清扫对提高患者总生存率及无病生存率具有重要意义。Lardinois等^[62]^研究也认为对于临床Ⅰ期NSCLC进行系统性纵隔淋巴结清扫可明显提高患者术后无病生存率及减低术后局部复发率，同时该研究还认为系统性淋巴结清扫与系统性淋巴结取样相比，并不增加患者围手术期并发症发生率及住院时间。

然而，ACOSOG Z0030实验组（American College of Surgery Oncology Z0030 Trial）对1, 023例诊断为T1-2N0-1期的NSCLC患者进行随机对照研究^[63]^报道：498例行纵隔淋巴结取样，5年无病生存率为68%；525例行系统性淋巴结清扫，5年无病生存率为69%。两组患者术后局部复发率及远处转移复发率无差异。因此，ASOSOG Z0030实验组专家认为对于术中系统性行肺门淋巴结及纵隔淋巴结取样未发现淋巴结转移的早期肺癌患者，系统性淋巴结清扫对改善患者生存率无统计学意义，其研究还发现两组对比局部复发率、区域复发率及远处转移复发率均无统计学意义，其*P*值分别为0.52、0.10及0.76。Sugi等^[64]^和Izbicki等^[65]^研究也发现对于T1期NSCLC行系统性淋巴结清扫或淋巴结取样，对患者5年生存率无明显影响。

选择性纵隔淋巴结清扫是根据肺癌原发病灶部位，清扫相应区域纵隔淋巴结及其周围组织。这一手术方式既避免了纵隔淋巴结取样时遗漏部分隐匿性纵隔淋巴结的弊端，又减少了纵隔淋巴结清扫程度，因而可缩短手术时间和减少术中及术后并发症，同时不影响患者术后总体生存率及无病生存率。近年来，国内外学者就肺癌手术中系统性纵隔淋巴结清扫与选择性纵隔淋巴结清扫进行了大量的研究，部分研究结果如表 1所示。

**1:** 探讨非小细胞肺癌淋巴结清扫程度的主要临床研究 Major clinical studies of the extent of lymph node dissection for non-small cell lung cancer

Author, year, country	Study type	Number of patient	Outcome	Results	Prognostic relevance
Ishiguro *et al*, 2010, Japan^[54]^	RS	772	5-year survival	SLND=76% CMLND=71.9%	No significance (*P*=0.29)
Darling *et al*, 2011 USA^[63]^	RCTs	1, 111	5-year DFS	MLNS=69.0% CMLND=68.0%	No significance (*P*=0.92)
Wu *et al*, 2001, China^[61]^	RCTs	532	5-year survival	MLNS=57.5% CMLND=82.2%	Better survival (*P*=0.01)
Lardinois *et al*, 2005, Switzerland^[62]^	RCTs	100	Median DFS	MLNS=44.8 month CMLND=60.2 month	Better DFS (*P* < 0.03)
Okada *et al*, 2006, Japan^[55]^	RCTs	377	5-year survival 5-year DFS	SLND=83.2% CMLND=79.2% SLND=76.4% CMLND=73.4	No significance (*P*=0.37) No significance (*P*=0.06)
Ma *et al*, 2013, China^[66]^	RCTs	96	5-year survival 5-year DFS	SLND=70.9% CMLND=65.5% SLND=64.0% CMLND=60.0%	No significance No significance
Ma *et al*, 2008, China^[53]^	RCTs	105	2 cm < T < 3 cm 5-year survival 5-year DFS T≤2 cm 5-year survival 5-year DFS	MLNS=55.8% SLND=81.6% MLNS=52.5% SLND=77.9%	Better survival (*P*=0.04) Better survival (*P*=0.03) No significance No significance
Sugi *et al*, 1998, Japan^[64]^	RCTs	115	5-year survival	MLNS=84% CMLND=81%	No significance
Doddali *et al*, 2005, France^[67]^	RS	465	5-year survival	MLNS=59.1% CMLND=64.7%	No significance (*P*=0.11)
RS: retrospective study; DFS: disease free survival; RCTs: randomized controlled trials; CMLND: complete mediastinal lymph node dissection; SLND: selective lymph node dissection; MLNS: mediastinal lymph node sampling.					

Okada等^[55]^研究证明临床Ⅰ期NSCLC行根治性纵隔淋巴结清扫与选择性纵隔淋巴结清扫对比，并不能改善患者总体生存率及无病生存率，而且术后局部及远处肿瘤复发率与选择性纵隔淋巴结清扫无差别。

综上所述，为减轻患者手术损伤及痛苦，选择性纵隔淋巴结清除有可能取代既往的系统性纵隔淋巴结清除，成为部分T1期NSCLC纵隔淋巴结清扫的可接受的清扫方式。但目前还缺乏随机分组研究的有力证据，未来期待更多研究证明依据术中冰冻检查特异性引流区淋巴结（肺门+肺叶特异纵隔引流区）是否转移来决定清扫方式：当特异性引流区淋巴结转移阳性时行系统性淋巴结清扫，若为阴性则行选择性淋巴结清扫，从而有效避免临床Ⅰ期肺癌过度淋巴结清扫，并可减少围手术期并发症和保护患者术后免疫功能^[54]^。
